# Running Shoe Recommendations Based on Gait Analysis Improve Perceptions of Comfort, Performance and Injury Risk: A Single‐Blind Randomised Crossover Trial

**DOI:** 10.1155/tsm2/1419641

**Published:** 2025-12-08

**Authors:** Andrew Fife, Jean-Francois Esculier, Codi Ramsey, Kim Hébert-Losier

**Affiliations:** ^1^ Division of Health, School of Sport & Human Movement, University of Waikato, Tauranga, New Zealand, waikato.ac.nz; ^2^ Research & Development, The Running Clinic^TM^ , Lac-Beauport, Québec, Canada; ^3^ Department of Physical Therapy, University of British Columbia, Vancouver, British Columbia, Canada, ubc.ca; ^4^ Institute of Sport, Exercise and Health, Otago Polytechnic, Dunedin, New Zealand

**Keywords:** deception, footwear, gait analysis, prescription, running

## Abstract

**Objectives:**

We examined how shoe recommendation based on gait analysis influences subjective perceptions of comfort, performance and injury reduction in runners while monitoring spatiotemporal and kinematic parameters.

**Design:**

Single‐blind crossover randomised controlled trial with repeated measures.

**Method:**

Twenty‐one women runners completed a clinical gait analysis and four 5‐min treadmill trials at a self‐selected comfortable speed sequentially in their own shoes (OS), the first experimental shoes (randomised), their OS, and the second experimental shoes (randomised). The two experimental shoes were identical except for their colour (randomised) and were presented to runners as either a ‘basic’ shoe or, deceptively, a ‘gait‐matched’ shoe selected for them based on the clinical gait analysis conducted.

**Results:**

Running Comfort Assessment Tool (RUN‐CAT) scores and 100 mm visual analogue scale ratings of subjective comfort, performance and injury reduction differed significantly between own and experimental shoes (*p*  <  0.001). Post‐hoc comparisons revealed that runners’ OS were the most comfortable (83.3 ± 3.8 mm) followed by gait‐matched (66.1 ± 21.5 mm) and then basic (49.0 ± 24.1 mm) shoes. RUN‐CAT, performance and injury reduction ratings were similar between own and gait‐matched shoes, but gait‐matched shoes had better mean difference (95% confidence intervals), RUN‐CAT (15.6 mm [5.7, 25.5]), performance (17.1 mm [5.6, 28.6]) and injury reduction (30.1 mm [8.9, 51.2]) scores than the basic shoes. Discrete spatiotemporal, foot strike angle and resultant tibial acceleration parameters were not significantly different between shoes (*p* ≥ 0.157). Most runners overall preferred their OS (71.4%), followed by gait‐matched (23.8%) and basic (4.8%) shoes.

**Conclusions:**

Shoe recommendation and description can significantly affect subjective shoe comfort and overall preferences without significantly altering spatiotemporal and kinematic parameters. Runners should be cautious while choosing shoes based on recommendations and descriptors derived from gait analysis or based solely on perceived comfort as runners’ subjective perceptions can be artificially manipulated.

**Trial Registration:** Australian New Zealand Clinical Trials Registry: ACTRN12623000516684.

## 1. Introduction

Many runners use shoes to increase comfort and reduce incidence of running‐related injuries [1]. The belief that shoes can reduce injury in part stems from marketing claims that individualised shoe prescription improves running mechanics [2]. Researchers have challenged the efficacy of shoe prescription practices for over a decade [3] and more recently concluded that the role of running shoe technology in injury reduction is likely overrated [4]. In spite of the evolution of shoe prescription theories and paradigms over the years (e.g. pronation control, impact force modification, habitual joint path and comfort filter), there is no compelling evidence to support current running shoe prescription methods (refer to Agresta, Giacomazzi, et al. [5] for a comprehensive review).

Runners currently seek shoe selection advice from many sources including running stores, friends and family, healthcare professionals and experts within the running community [6, 7]. While runners and researchers often prioritise reducing running‐related injury through shoe selection [6, 8], the link between running shoe technology and injury is poorly supported. In spite of industry and academic experts suggesting certain shoe properties based on the running level, the effectiveness of these recommendations is unknown [9], and these recommendations did not include the opinion of runners. Runners consider salespeople as experts [1], although salespeople may have beliefs that do not align with scientific evidence [10]. Runners purchasing shoes at specialty stores may undergo a gait analysis, which sometimes increases the trust some of these runners have in the expertise of salespeople [1], which in turn plays an important role in runners’ shoe selection [7]. However, at other times, runners perceived the so‐called expertise of salespeople more negatively as a sales tactic and gimmick [11]. A gait analysis conducted in specialty running stores typically involves running in front of a salesperson who attempts to determine individual running biomechanics and prescribe the ‘perfect shoe’ [12]. It is also a common practice to assess foot shape and prescribe a shoe on that basis [2] in spite of the lack of evidence to support the view that this practice prevents running‐related injury.

Comfort is consistently prioritised during running shoe selection [7, 13] and can be influenced by numerous factors including shoe construction and individual anatomy [14]. Comfort is also manipulable. For example, runners rated footwear comfort differently for identical shoes based on product descriptions regarding price, market availability and comfort [15]. In spite of widespread use of gait analysis in retail settings, little is known about how such personalised recommendations affect runners’ perceptions of comfort and subsequent shoe choice, irrespective of whether the shoe ‘matched’ an individual or not.

To our knowledge, no study has directly examined if recommending a shoe based on gait analysis alters runners’ footwear perceptions, irrespective of whether the shoe is ‘matched’ to the person or not. Therefore, we aimed to determine the influence of expert recommendation based on gait analysis on perceptions of subjective comfort, performance and injury reduction in runners while monitoring spatiotemporal and kinematic parameters. We hypothesised that runners would score and rank gait‐matched shoes more favourably than basic shoes. A secondary aim was to assess the between‐trial reliability of the studied measures based on repeat measure from runners running in their own shoes (OS). Our findings may help clarify the actual influence of shoe prescription methods on footwear selection, therefore empowering runners to make a more evidence‐based footwear selection.

## 2. Materials and Methods

### 2.1. Participants

A sample size of 19 participants was required based on a priori calculations performed in G∗Power 3.1.9.7 to achieve a power of β = 0.80 with an *α* = 0.05 and ability to detect a *moderate* difference (Cohen’s *d* = 0.70, two‐tail) between two dependent means. This calculation was based on the *moderate* difference detected in comfort scores from a study of similar design [15], assuming superior comfort in gait‐recommended shoes. To account for potential missing data (10%), 21 participants were targeted and data collection ceased once sample size was reached.

This study was part of a larger project examining factors that influence the selection of running footwear and subjective perceptions [16]. Participants for this study were recruited in May 2023 through online advertisements within the running community and posters placed at the University of Waikato campus and in local running stores in the Bay of Plenty area, New Zealand. Eligibility requirements included: (1) women aged 18 years and older and (2) running a minimum of once per week for at least 1 month. Women were targeted because historically they were relatively underrepresented in sport and exercise sciences [17], including in running footwear research [18]. Potential participants were excluded if they were injured within the previous month based on a consensus definition [19]. The Human Research Ethics Committee of the University of Waikato (HREC(HECS)2023#11) approved the trial, which was registered on the Australian New Zealand Clinical Trials Registry on 19 May 2023. Pre‐registration was sought (submitted 26 April 2023, before first participant enrolment), but because of time constraints and delays in receiving approval, the first participant was enrolled on 5 May 2023 (i.e. 12 days before approval), with the last participant completing the study on 20 May 2023. The trial did not deviate from the original plan. Participants received an information sheet detailing the participation requirements, benefits and risks, and signed an informed consent document prior to participating. We followed the reporting guidelines stated in the CONSORT for randomised controlled trials 2025 checklist [20]. Participants were not involved in the design or reporting of the trial results.

### 2.2. Design

**Figure 1:**
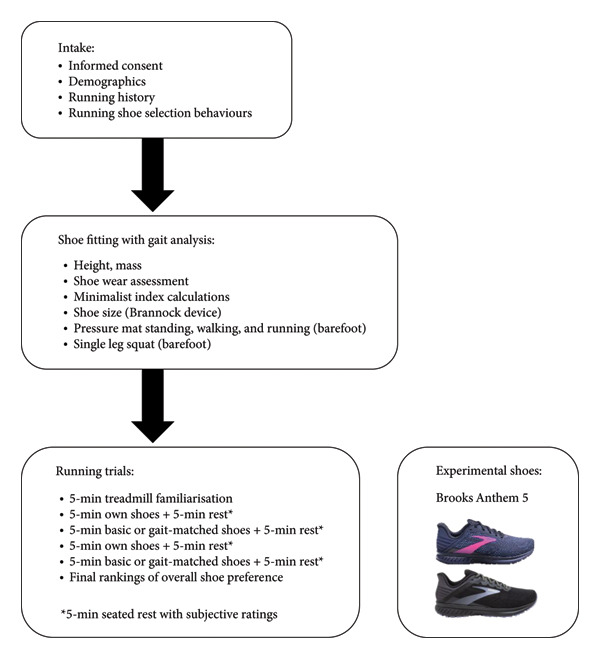
Flow diagram of the experimental design and image of the experimental shoes.

A single‐blind crossover randomised control trial was conducted (Figure 1) based on a superiority framework, expecting participants would prefer the gait‐matched shoes over the basic ones. Evaluations were performed during a single 90‐min session at the University of Waikato Adams Centre for High Performance, New Zealand, and included three stages: intake, shoe fitting with gait analysis and running trials. During running trials, participants ran in their OS and two experimental shoes: ‘basic’ or ‘gait‐matched’ shoes. Both experimental shoes were Brooks Anthem 5 (WA, USA) available in two colours (black and blue) and three sizes (US 7.5, 8.5, 9.5 Women). Participants were led to believe gait‐matched shoes were prescribed to them based on the shoe fitting and gait analysis process, matching their foot shape and running style to the shoe. The following standardised descriptions of both experimental shoes were verbally provided to runners immediately before running in them:

• ***Basic:*** ‘This shoe model is quite basic. It is a generic shoe that doesn’t necessarily match your foot shape or running style. Basically, it can be used for distance running, but it is not specifically suited to you’.
• ***Gait-matched:*** ‘Based on all the tests we did and the six pairs of shoes we have in the lab, this pair is the best option for you to maximise your comfort. Since this shoe matches your foot shape and running style, it will be extra comfortable when you run. We know from research that when a shoe is super comfortable and matches your body and running style, it usually leads to better performance and lower injury risk’.

No participant was harmed during experimentation or reported experiencing an injury or delayed onset muscle soreness post‐participation.

### 2.3. Outcome Measures

Primary outcomes included scores from the Running shoe Comfort Assessment Tool (RUN‐CAT) [21] and subjective perceptions of comfort, performance and injury reduction using 100 mm visual analogue scales (VAS). Secondary outcomes included other subjective perception scores (i.e. how much the shoe matches individual running style, running difficulty and running pleasure) using 100 mm VAS [22], overall shoe rankings and biomechanical measures (i.e. flight time, contact time, cadence, duty factor (DF), foot strike angle and resultant tibial acceleration) described in detail below.

### 2.4. Experimental Protocol

#### 2.4.1. Intake

Before experimentation, data on demographics and running history were collected using an intake survey. A wall‐mounted tape measure and Wedderburn WM202 scale (NSW, Australia) were used to record body height and mass of participants barefoot to the nearest 0.1 cm and 0.1 kg, respectively. As part of the intake survey, runners ranked their personal running motivations from most to least important using a list adapted from the motivation of marathon scale [23, 24]. Runners also ranked their running footwear priorities (i.e. comfort, injury reduction and performance). Runners reported their sources of advice regarding running footwear and factors that influence their running shoe selection and ranked them in order of importance. Runners also reported their typical running shoe purchase locations.

#### 2.4.2. Shoe Fitting With Gait Analysis

The primary investigator (AF) conducted the shoe fitting with gait analysis for all participants to replicate practices commonly found in specialty running shoe stores [12]. This investigator had spent an extensive period in various speciality running stores observing and interacting with footwear salespeople prior to this study. The investigator also had prior experience working in sports stores as a retail consultant.

The shoe fitting began with AF assessing the wear and minimalist index [25] of runners’ OS. A Brannock device (NY, USA) was then used to measure foot size and match runners with the closest available lab‐provided shoe size.

The gait analysis began by using an AmCube pressure plate (ZA des Moulins, France) to examine runners’ barefoot foot shape in standing and pressure distribution during walking and running barefoot, striking the pressure plate. Following the pressure plate activities, runners performed a single‐leg squat barefoot on each leg while the primary investigator watched their lower limb kinematics. Throughout these activities, no data were formally collected as the process was only performed to simulate in‐store experiences and increase credibility in the gait‐matched recommendations. The act of ‘matching’ was part of the experimental deception. To assist in convincing participants that their gait‐matched shoes were specifically matched to them, AF simulated looking closely at the data and results, recording ‘fake’ notes using pen and paper. If participants commented on their own foot type or gait patterns during the process, AF would acknowledge their comments with statements and verbal intonations such as ‘I see’ or ‘uh‐um’.

#### 2.4.3. Running Trials

Following the shoe fitting with gait analysis, runners put their OS on and were instrumented with Blue Trident V2 inertial measurement unit (IMU) sensors (Vicon, Oxford, UK) on each ankle above the medial malleolus on the anteromedial aspect of the distal tibia [26, 27], secured using the manufacturer’s straps and athletic tape. The *y*‐axis of the IMU was aligned with the long axis of the tibia, and *x*‐axis was aligned with the anterior‐posterior running direction. All running trials were completed on a motorised treadmill (Steelflex PT10 Fitness; Steelflex Fitness, Taipei, Taiwan, China). A tablet with a high‐definition camera (Apple Inc., USA) was placed 1.5 m from the treadmill to record sagittal plane foot strike angles of the left leg in each footwear condition at 240 Hz. A 1‐m Optojump Next (Microgate, Bolzano, Italy) modular system with infrared light sensors was placed parallel to the treadmill and used to record spatiotemporal parameters at 1000 Hz.

In total, participants completed four 5‐min running trials after running an initial 5 min in their OS for familiarisation [28]. During familiarisation, runners were instructed to run at a self‐selected pace that they could comfortably sustain for 20 min [29]. The primary investigator slowly increased treadmill speed upon verbal consent from runners until the desired speed was obtained [15]. The final minute of the familiarisation period was used to confirm the self‐selected speed that would be used for all subsequent running trials. Throughout this process and running trials, treadmill speed was blinded to participants. Runners completed the first 5‐min running trial in their OS immediately following familiarisation (i.e. no rest). Runners used their OS during the first and third running trials to act as a control and measure intra‐session reliability of measures. The second and fourth running trials were randomised and completed wearing either the ‘basic’ or ‘gait‐matched’ shoes. Experimental shoe order and colour were allocated using a random number generator online by the primary investigator (AF). Biomechanical data (Optojump, IMU, and foot strike data) were collected for 30 s from the fourth minute of each trial. Runners rested 5 min between trials seated with footwear removed and all shoes out of sight. During this time, runners completed surveys via the XM Qualtrics software (https://www.qualtrics.com) on subjective footwear perceptions.

The surveys taken after each running trial examined subjective footwear perceptions relating to overall comfort, performance and injury reduction, as well as how much the shoe matched their individual running style, running difficulty and running pleasure using 100 mm VAS [30, 31]. The VAS included anchors like ‘much worse (0), neutral/uncertain (50) and much improved (100)’ and were colour‐coded across questions for congruence, which have shown *fair* to *good* test–retest reliability (intraclass correlation coefficients (ICC) 0.66 to 0.85, typical error 7.6–10.7 mm) [31]. In addition, RUN‐CAT was calculated based on VAS ratings of heel cushioning, forefoot cushioning, forefoot flexibility and overall stability [21], with Goldilocks anchors like ‘heel not cushioned enough (0), ideal (50), heel cushioned too much (100)’ that were also colour‐coded. Ratings were converted to a final RUN‐CAT score where 0 represents the least ideal and 100 represents the most ideal shoe properties following validated and reliable methods [21]. After the final running trial, runners completed a final survey ranking their own, gait‐matched and basic shoes based on overall preference, best‐matched to their individual running style, performance, injury reduction and comfort. All survey questions are provided in Supporting Information. Participant blinding to the footwear conditions and study intent was assessed verbally at the end of the laboratory session.

### 2.5. Data Processing

The 1‐m Optojump data were averaged across each 30‐s running trial to increase representativeness of the running gait of participants (80–85 steps per trial) [30, 32]. DF was determined using Optojump data and the following calculations [30]: DF = ((SF x *t*_*c*_)/2)*x* 100%, where SF is stride frequency and t_c_ is contact time. Raw IMU data were filtered using an 100 Hz low‐pass bidirectional fourth order Butterworth filter [26] applied using Visual 3D Professional™ (version 2023.01.4, C‐Motion Inc., Germantown, Maryland, USA). The resultant tibial acceleration was calculated as √(*x* ^2^ + *y* ^2^ + *z* ^2^). Peak resultant tibial acceleration was selected over vertical tibial acceleration because it is less susceptible to sensor misalignment and is more reliable [33]. Furthermore, the resultant tibial acceleration shows weak‐to‐moderate correlations with peak tibial force during running, whereas vertical tibial acceleration does not [34]. Foot strike angle was measured using Onform (CO, USA) video analysis software, and foot strike pattern was visually interpreted as rearfoot, midfoot or forefoot following methods described elsewhere [35]. One foot strike from the middle of the 30 s video with a clearly defined ground contact in the middle portion of the screen was used for this purpose.

### 2.6. Statistical Analysis

Survey data were imported into Microsoft Excel (version 2302 Build 16.0.16130.20298) and analysed by shoe condition (own, basic, gait‐matched) using IBM SPSS Statistics [version 29.0.0.0 (241)] software. Means with standard deviations and counts with percent values were used to describe the data. Reliability was assessed using subjective VAS scores and biomechanical data from the first and second trial of runners in their OS. ICC_3,*k*_ estimates and their 95% confidence intervals (lower, upper) were calculated using SPSS based on mean rating, absolute‐agreement, two‐way mixed‐effects models and interpreted using values < 0.50 as *poor*, from 0.50 to 0.75 as *moderate*, from 0.75 to 0.90 as *good* and > 0.90 as *excellent* absolute reliability [36]. The standard error of measurements (SEM) and coefficient of variation (CV) were calculated using $SEM=SD\sqrt{1-ICC}$ and *C* *V* = (*S* *E* *M*/*M* *e* *a* *n* (*a* *l* *l* *d* *a* *t* *a*)) *x* 100%, and level of errors was deemed acceptable when CV < 10% [37].

To examine between shoe differences, one‐way ANOVA with repeated measures were conducted on subjective and biomechanical measures. The two trials ran in OS were averaged for comparative analyses, resulting in three shoe comparisons: own, basic and gait‐matched. In the presence of a main effect of shoe, post‐hoc Bonferroni‐adjusted pairwise comparisons were performed. Mean differences between shoe conditions were calculated with their 95% confidence intervals. Effect sizes were reported using partial eta squared (η^2^) with values reaching 0.01 considered *small*, 0.06 considered *medium*, and 0.14 considered *large* in magnitude [38]. Independent *t*‐tests were used to compare the minimalist index of participants’ OS to the experimental shoes. In all analyses, *p*  <  0.05 was considered statistically significant. In presence of missing data, all available data were used for analysis using a pairwise deletion approach.

## 3. Results

### 3.1. Descriptive Data

Twenty‐one runners were recruited, started the experiment, and completed the study (Table 1). Most runners had over 3 years of running experience and an average self‐reported 5‐km best time of 25:30 ± 4:42 min in the previous year. Nearly all runners (*n* = 19, 90.5%) exhibited a rearfoot foot strike pattern using their OS. The minimalist index of participants’ OS (31.6 ± 11.2) was not significantly different from the experimental shoes (28.0 ± 0.0, *p* = 0.156). The average treadmill running speed was 8.6 ± 1.5 km/h (range: 6.1–11.3 km/h).

**Table 1:** Summary of the characteristics, running experience, running characteristics and shoe properties of the participating women runners (*n* = 21).

Characteristics	
Age (years)	42.9 ± 7.7
Height (cm)	166.3 ± 6.5
Mass (kg)	59.9 ± 6.8
Body mass index (kg/m^2^)	21.7 ± 2.8
Ethnicity	
New Zealand European	17 (81.0%)
European	2 (9.5%)
Asian	1 (4.8%)
Māori	1 (4.8%)
South African	1 (4.8%)
Running history	
Between 0 and 3 years	3 (14.3%)
More than 3 years	18 (85.7%)
Years running	12.3 ± 9.1
Race competitively (yes)	7 (33.3%)
5 km time (mm:ss)	25:30 ± 4:42
Weekly sessions	3.4 ± 1.4
Weekly distance (km)	46.1 ± 26.9
Own shoe foot strike pattern	
Rearfoot	19 (90.5%)
Midfoot	1 (4.8%)
Forefoot	1 (4.8%)
Minimalist index (%)^a^	
Experimental shoes	28.0 (0.0%)
Own shoes	31.6 (11.2%)
Experimental shoe size	
US 7.5	10 (47.6%)
US 8.5	7 (33.3%)
US 9.5	4 (19.0%)
Self‐selected treadmill speed (km/h)	8.6 (1.5)

*Note*: Data are means ± standard deviations or counts (percentages).

^a^Minimalist index range: 0% (lowest) to 100% (highest) degree of minimalism.

**Figure 3:**
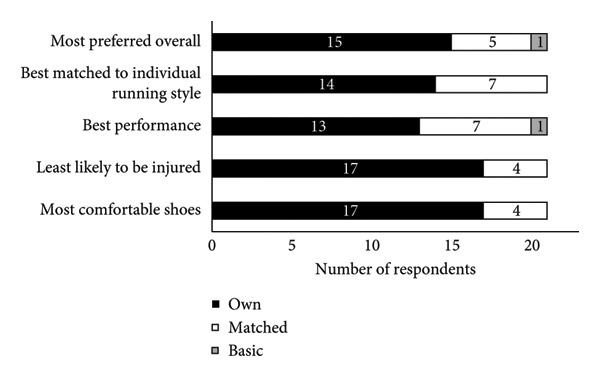
Runners ranked their own shoes, gait‐matched shoes and basic shoes against each other in a head‐to‐head comparison. The numbers represent the runners who selected the shoes as their most preferred option for a given criterion.

A summary of running shoe selection behaviours of participants is presented in Figures 2 and 3. When choosing running shoes, runners most frequently prioritised comfort (23.8%), gait analysis findings (19.0%) and shoe specifications and technologies (19.0%, Figure 2(a)). To inform their shoe selection, runners most frequently prioritised advice from running shoe stores in‐person (42.9%), friends (19.0%) and coaches (14.3%, Figure 2(b)). Primary motivations for running included enjoyment (38.1%), general health (28.6%) and team affiliation (14.3%, Figure 3a). Regarding footwear design, injury reduction (52.4%) was most important to runners, followed by comfort (38.1%) and performance (9.5%, Figure 3b). Runners most often purchased their shoes in specialty running stores (47.6%, Figure 2(e)).

Figure 2(a) Main factors that influenced shoe selection, (b) main sources of footwear recommendations, (c) main motivation for participation in running as a sport, (d) relative importance of shoe design priorities and (e) locations where runners typically purchased their running shoes. Participants selected and ranked factors, sources of advice, motivations for running and shoe design priorities in order of importance. Percentages are based on the sample size of 21 runners, although participants were able to select more than one option. Abbreviations: HCP, healthcare provider.

**Figure:**
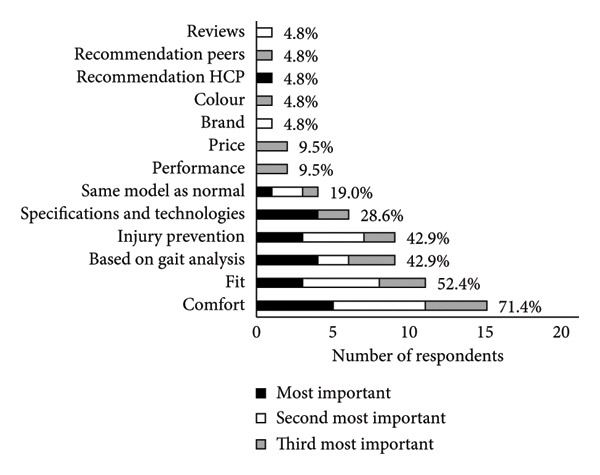
(a)

**Figure:**
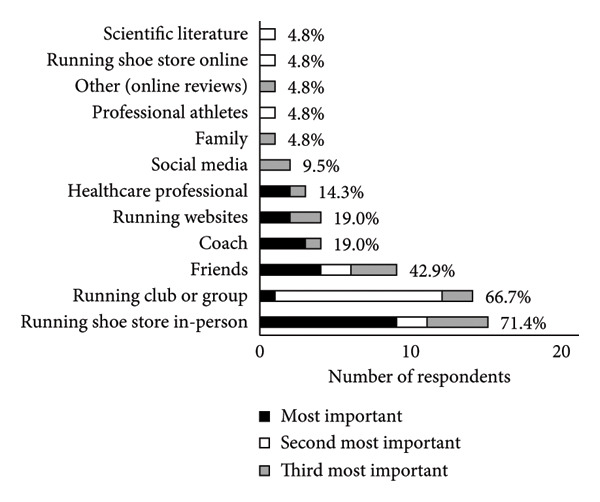
(b)

**Figure:**
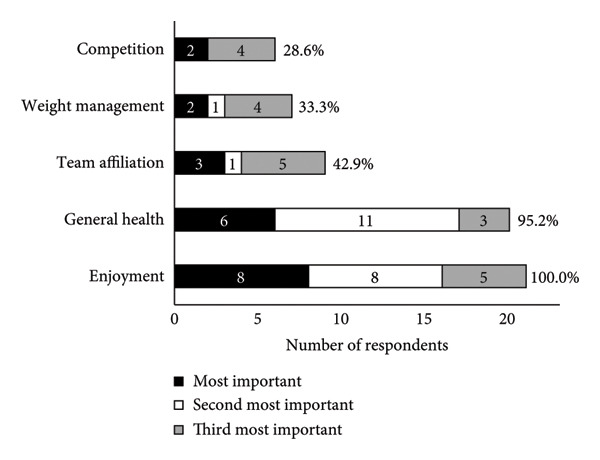
(c)

**Figure:**
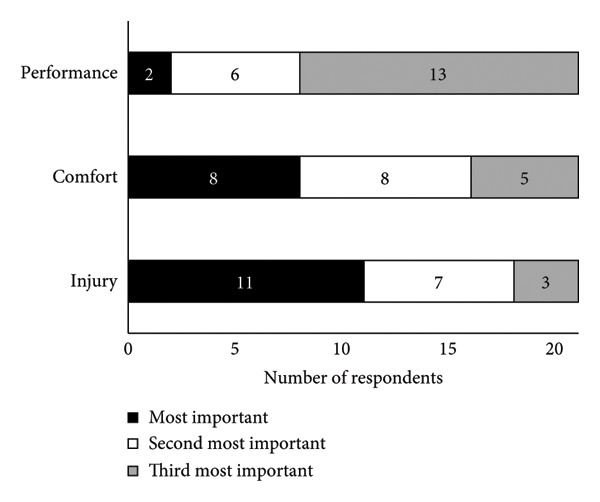
(d)

**Figure:**
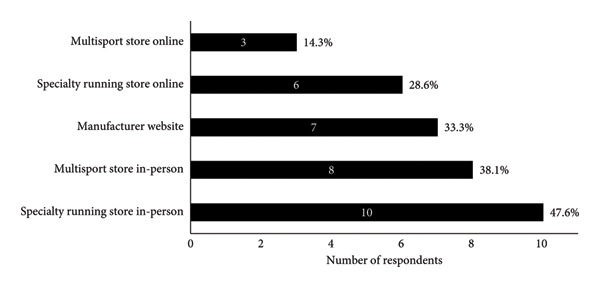
(e)

### 3.2. Reliability

Of the subjective measures collected, VAS scores for RUN‐CAT, performance, how much the shoe matches individual running style and running pleasure demonstrated *good* reliability (Table 2). Perceptions of overall comfort, injury risk reduction and running difficulty demonstrated *moderate* reliability. Most subjective measures exhibited acceptable errors (CV ≤ 9.8%, SEM ≤ 7.2 mm), with higher errors for perceived injury risk reduction (CV 17.0%, SEM 11.0 mm) and running difficulty (CV 15.4%, SEM 11.0 mm). All biomechanical measures showed *excellent* reliability (ICC ≥ 0.954) and acceptable levels of errors (CV ≤ 9.2%), except for resultant tibial acceleration where reliability was *good* and errors were above 10% (ICC = 0.824, CV 11.5%).

**Table 2:** Reliability of subjective perceptions and biomechanical measures of participating women runners (*n* = 21) for the two trials ran in their own shoes.

Item	Own shoes 1	Own shoes 2	Δmean	ICC [95% CI]	SEM [95% CI]	CV (%) [95% CI]
Subjective VAS scores (100 mm scale)^a^						
RUN‐CAT	82.7 ± 14.3	79.9 ± 16.7	2.8 ± 0.2	0.877 [0.696, 0.951]	5.4 [3.4, 8.5]	6.9 [4.4, 10.9]
Overall comfort	85.9 ± 7.3	81.7 ± 11.1	4.2 ± 8.5	0.700 [0.273,0.877]	5.2 [3.3, 8.1]	6.2 [4.0, 9.6]
Performance	67.6 ± 15.4	64.7 ± 18.3	2.9 ± 11.8	0.861 [0.663, 0.943]	6.3 [4.0, 9.7]	9.5 [6.1, 14.7]
Injury risk reduction	64.1 ± 19.6	65.0 ± 24.4	−0.9 ± 20.2	0.747 [0.366, 0.898]	11.0 [7.0, 17.4]	17.0 [10.8, 26.9]
Matches running style	72.1 ± 17.3	75.0 ± 17.5	−2.8 ± 13.4	0.826 [0.577, 0.929]	7.2 [4.6, 11.2]	9.8 [6.3, 15.3]
Difficulty (easier‐harder)	70.6 ± 18.7	71.5 ± 17.5	−0.9 ± 19.1	0.624 [0.050, 0.849]	11.0 [6.9, 17.4]	15.4 [9.8, 24.5]
Pleasure	85.1 ± 10.5	81.4 ± 12.7	3.7 ± 8.2	0.842 [0.599, 0.937]	4.6 [2.9, 7.4]	5.6 [3.5, 8.9]
Biomechanical measures						
Flight time (ms)	56.0 (22.9)	53.3 (23.9)	2.6 ± 9.3	0.954 [0.885, 0.981]	5.0 [3.2, 8.0]	9.2 [8.9, 14.6]
Contact time (ms)	314.5 (36.1)	317.0 (37.6)	−2.5 ± 9.6	0.982 [0.955, 0.992]	4.9 [3.3, 7.8]	1.6 [1.0, 2.5]
Cadence (steps/min)	166.0 (9.5)	165.0 (9.1)	0.0 ± 1.7	0.991 [0.978, 0.997]	0.9 [0.5, 1.4]	0.5 [0.3, 0.8]
Duty factor (%)	0.4 (0.0)	0.4 (0.0)	0.0 ± 0.0	0.962 [0.909, 0.985]	0.0 [0.0, 0.0]	1.6 [1.0, 2.4]
Foot strike angle (°)	10.4 (7.8)	10.5 (7.7)	−0.1 ± 2.1	0.982 [0.955, 0.993]	1.0 [0.6, 1.6]	8.3 [5.2, 13.1]
Tibial resultant acceleration (g)	9.0 (2.3)	8.9 (2.3)	0.1 ± 0.7	0.824 [0.541, 0.933]	1.0 [0.6, 1.7]	11.5 [7.1, 18.5]

*Notes:* Data are means ± standard deviations or estimates with 95% confidence intervals [lower, upper].

Abbreviations: Δmean, mean difference; CI, confidence interval; CV, coefficient of variation; ICC, intraclass correlation coefficients; RUN‐CAT, running shoe comfort assessment tool; SEM, standard error of measurement; VAS, visual analogue scale.

^a^Higher scores reflect more positive outcomes where 100 reflects most ideal shoe properties for RUN‐CAT, most comfortable imaginable, much improved performance, much lower injury risk, matches running style very well, much easier and very pleasant.

### 3.3. Subjective Measures

There was a significant (*p* ≤ 0.001) and *large* ($\eta_{\text{p}}^{2}$ 0.362–0.565) main effect of shoe on all subjective perception measures (Table 3). Post‐hoc testing identified that runners were overall more comfortable and found running more pleasurable in their OS followed by the gait‐matched shoes, and rated basic shoes the lowest. There were no significant differences in the remaining subjective measures (i.e. performance, injury reduction, match to running style and running difficulty) between own and gait‐matched shoes, but scores were significantly lower for the basic shoes.

**Table 3:** Summary of subjective perceptions and biomechanical measures of participating women runners (*n* = 21), along with between shoe comparisons.

Item	Mean ± SD Δmean [95% CI]	ANOVA *p* value, $\eta_{\text{p}}^{2}$ (*magnitude*) Pairwise comparison *p* value
*Subjective VAS scores (100 mm scale)* ^*a*^		
RUN‐CAT	*n* = 20	Shoe *p* < 0.001^∗^, $\eta_{\text{p}}^{2}$ = 0.371 (*large*)
OS	81.3 ± 14.7	OS*-* N_b_ *p* = 0.005^∗^
N_b_	61.2 ± 18.3	OS‐N_m_ *p* = 0.800
N_m_	76.9 ± 14.7	N_b_‐N_m_ *p* = 0.002^∗^
Shoe comparisons		
N_b_ *-*OS	−20.0 [−34.4, −5.7]	
N_m_ *-*OS	−4.4 [−14.6, 5.7]	
N_b_‐N_m_	−15.6 [−25.5, −5.7]	
Overall comfort		Shoe *p* < 0.001^∗^, $\eta_{\text{p}}^{2}$ = 0.530 (*large*)
OS	83.8 ± 8.3	OS*-* N_b_ *p* < 0.001^∗^
N_b_	49.0 ± 24.1	OS‐N_m_ *p* = 0.003^∗^
N_m_	66.1 ± 21.5	N_b_‐N_m_ *p* = 0.024^∗^
Shoe comparisons		
N_b_ *-*OS	−34.8 [−48.2, −21.4]	
N_m_ *-*OS	−17.7 [−29.6, −5.8]	
N_b_‐N_m_	−17.1 [−32.2, −2.0]	
Performance		Shoe *p* < 0.001^∗^, $\eta_{\text{p}}^{2}$ = 0.413 (*large*)
OS	66.1 ± 15.9	OS‐N_b_ *p* < 0.001^∗^
N_b_	37.9 ± 21.0	OS‐N_m_ *p* = 0.148
N_m_	55.0 ± 18.6	N_b_‐N_m_ *p* = 0.003^∗^
Shoe comparisons		
N_b_ *-*OS	−28.3 [−44.5, −12.1]	
N_m_ *-*OS	−11.2 [−25.2, 2.8]	
N_b_‐N_m_	−17.1 [−28.6, −5.6]	
Injury reduction		Shoe *p* < 0.001^∗^, $\eta_{\text{p}}^{2}$ = 0.362 (*large*)
OS	64.6 ± 19.7	OS*-* N_b_ *p* < 0.004^∗^
N_b_	34.5 ± 23.5	OS‐N_m_ *p* = 0.402
N_m_	54.6 ± 20.5	N_b_‐N_m_ *p* < 0.001^∗^
Shoe comparisons		
N_b_ *-*OS	−30.1 [‐51.2, −8.9]	
N_m_ *-*OS	−10.0 [−26.7, 6.7]	
N_b_‐N_m_	−20.1 [−31.1, −9.1]	
Matches running style		Shoe *p* < 0.001^∗^, $\eta_{\text{p}}^{2}$ = 0.551 (*large*)
OS	73.6 ± 16.1	OS*-* N_b_ *p* < 0.001^∗^
N_b_	37.0 ± 18.2	OS‐N_m_ *p* = 0.440
N_m_	65.9 ± 19.4	N_b_‐N_m_ *p* = 0.440^∗^
Shoe comparisons		
N_b_ *-*OS	−36.6 [−53.2, −20.0]	
N_m_ *-*OS	−7.6 [−20.9, 5.6]	
N_b_‐N_m_	−29.0 [−42.1, −15.8]	
Difficulty (easier‐harder) (mm)		Shoe *p* = 0.001^∗^, $\eta_{\text{p}}^{2}$ = 0.394 (*large*)
OS	71.0 ± 15.4	OS*-* N_b_ *p* < 0.001^∗^
N_b_	43.3 ± 18.9	OS‐N_m_ *p* = 0.061
N_m_	57.3 ± 19.2	N_b_‐N_m_ *p* = 0.033^∗^
Shoe comparisons		
N_b_‐OS	−27.7 [−42.9, −12.5]	
N_m_ *-*OS	−13.7 [−28.0, 0.5]	
N_b_‐N_m_	−14.0 [−27.0, −0.9]	
Pleasure		Shoe *p* = 0.001^∗^, $\eta_{\text{p}}^{2}$ = 0.565 (*large*)
OS	82.3 ± 10.9	OS*-* N_b_ *p* = 0.001^∗^
N_b_	44.7 ± 20.7	OS*-* N_m_ *p* = 0.009^∗^
N_m_	65.8 ± 21.6	N_b_‐N_m_ *p* = 0.004^∗^
Shoe comparisons		
N_b_ *-*OS	−38.5 [−52.1, −25.0]	
N_m_ *-*OS	−17.4 [−31.0, −3.9]	
N_b_‐N_m_	−21.1 [−35.9, −6.3]	

*Biomechanical measures*		
Tibial resultant acceleration (g)	*n* = 19	Shoe *p* = 0.009^∗^, $\eta_{\text{p}}^{2}$ = 0.232 (*large*)
OS	9.0 ± 2.7	OS*-* N_b_ *p* = 0.052
N_b_	9.8 ± 3.3	OS*-* N_m_ *p* = 0.011^∗^
N_m_	9.9 ± 3.3	N_b_‐N_m_ *p* = 1.000
Shoe comparisons		
N_b_ *-*OS	−0.8 [−1.6, 0.0]	
N_m_‐OS	−1.0 [−1.7, −0.2]	
N_b_‐N_m_	−0.2 [−1.1, 0.7]	
Flight time (ms)		
OS	52.0 ± 25.8	
N_b_	50.0 ± 29.5	
N_m_	49.7 ± 26.4	
Shoe comparisons		Shoe *p* = 0.421, $\eta_{\text{p}}^{2}$ = 0.042
N_b_ *-*OS	−2.0 [−7.7, 3.6]	
N_m_ *-*OS	−2.4 [−7.3, 2.6]	
N_b_‐N_m_	0.3 [−4.0, 4.6]	
Contact time (ms)		Shoe *p* = 0.623, $\eta_{\text{p}}^{2}$ = 0.023
OS	315.7 ± 37.4	
N_b_	315.3 ± 39.4	
N_m_	317.1 ± 38.4	
Shoe comparisons		
N_b_ *-*OS	−0.4 [−6.0, 5.2]	
N_m_ *-*OS	1.4 [−4.0, 6.8]	
N_b_‐N_m_	−1.8 [−5.6, 2.0]	
Cadence (steps/min)	*n* = 20	Shoe *p* = 0.157, $\eta_{\text{p}}^{2}$ = 0.093 (*medium*)
OS	165.0 ± 9.5	
N_b_	165.9 ± 9.0	
N_m_	165.4 ± 8.2	
Shoe comparisons		
N_b_ *-*OS	0.9 [−0.2, 1.9]	
N_m_ *-*OS	0.4 [−0.9, 1.6]	
N_b_‐N_m_	0.5 [−0.7, 1.7]	
Duty factor (%)		Shoe *p* = 0.524, $\eta_{\text{p}}^{2}$ = 0.032
OS	0.4 ± 0.0	
N_b_	0.4 ± 0.0	
N_m_	0.4 ± 0.0	
Shoe comparisons		
N_b_ *-*OS	0.0 [0.0, 0.0]	
N_m_ *-*OS	0.0 [0.0, 0.0]	
N_b_‐N_m_	0.0 [0.0, 0.0]	
Foot strike angle (°)		Shoe *p* < 0.001^∗^, $\eta_{\text{p}}^{2}$ = 0.356 (*large*)
OS	10.5 ± 7.6	OS*-* N_b_ *p* < 0.001^∗^
N_b_	12.6 ± 8.2	OS*-* N_m_ *p* = 0.055
N_m_	11.9 ± 9.1	N_b_‐N_m_ *p* = 0.182
Shoe comparisons		
N_b_ *-*OS	2.1 [0.9, 3.3]	
N_m_ *-*OS	1.4 [0.0, 2.8]	
N_b_‐N_m_	0.7 [‐0.2, 1.7]	

*Note*: Data are means ± standard deviations or mean differences with 95% confidence intervals [lower, upper]. One‐way ANOVA includes three shoe conditions: own shoes (OS), ‘basic’ shoes (N_b_) and ‘gait‐matched’ shoes (N_m_). Significant main effects were interpreted as *small*, *medium* and *large* when $\eta_{\text{p}}^{2}$ reached 0.01, 0.06 and 0.14, respectively, and further explored using post‐hoc pairwise comparisons. Δmean, mean difference; RUN‐CAT, running shoe comfort assessment tool;

Abbreviations: CI, confidence interval; CV, coefficient of variation; ICC, intraclass correlation coefficients; SD, standard deviation; SEM, standard error of measurement; VAS, visual analogue scale.

^a^Higher scores reflect more positive outcomes where 100 reflects most ideal shoe properties for RUN‐CAT, most comfortable imaginable, much improved performance, much lower injury risk, matches running style very well, much easier and very pleasant.

^∗^Statistically significant (*p*  <  0.05).

### 3.4. Biomechanical Measures

There was a significant and *large* ($\eta_{\text{p}}^{2}$ 0.232–0.356) main effect of shoe on resultant tibial acceleration (*p* = 0.009) and foot strike angle (*p*  <  0.001). Post‐hoc testing identified that runners exhibited lower acceleration in their OS compared to the gait‐matched shoes, and lower foot strike angles using their OS than basic shoes. There were no other significant differences between shoes for biomechanical measures (Table 3).

### 3.5. Overall Preference

When ranking shoe preferences, runners consistently preferred their OS followed by the gait‐matched shoes across all subjective categories (i.e. overall, comfort, performance, injury reduction and matched to individual running style), while basic shoes were the least preferred (Figure 3). For overall shoe preference, 15 runners (71.4%) preferred their OS, 5 runners (23.8%) the gait‐matched shoes and one runner (4.8%) the basic shoes. No participant indicated that they believed the experimental shoes to be identical when assessed for blinding effectiveness.

## 4. Discussion

This is the first study to our knowledge that assessed how deceptively recommending a shoe based on gait analysis influences subjective perceptions of comfort, performance and injury reduction. Our hypothesis that runners would rate shoes matched to their gait more favourably than basic shoes was supported. Runners rated gait‐matched shoes significantly more favourably than basic shoes in every subjective measure, and similarly to their OS in all measures except for overall comfort and running pleasure. Gait‐matched shoes were consistently rated over 10 mm higher than basic shoes on VAS, indicating a clinically meaningful difference [39]. Indeed, all VAS differences between the experimental shoes (range 14.0–29.0 mm) exceeded the SEM values (range 4.6–11.0 mm) we identified in our reliability analysis, with the largest difference being for the VAS on how well the shoes matched their running style.

The preference of our runners for the deceptively described gait‐matched shoes over the basic shoes further support the idea that comfort can be influenced through psychological means. Previous research has demonstrated that participants informed about mass differences between basketball shoes jump higher and shuffle cut better compared to blinded counterparts [40]. Furthermore, shoes from popular brands with favourable public images are preferred over generic counterparts [41]. Even description of running shoes based on price, material and market availability can influence perceived comfort of footwear [15]. From a psychological lens, it is possible that runners modified their perceptual ratings based on what they believed we (the researchers) wanted to hear, in line with the Hawthorne effect [42]. Marketing‐based expectations (e.g. price, brand) generate expectancy effects that can influence behaviours and sensory outcomes [43]. Runners likely had elevated expectations regarding the gait‐matched shoes linked with the shoe‐fitting process, leading to higher subjective ratings and a ‘marketing placebo effect’ [43] biasing subjective ratings in spite of the basic and ‘gait‐matched shoes being structurally identical.

The subjective preferences for gait‐matched shoes were not associated with significant changes in running spatiotemporal and kinematic parameters. While resultant tibial acceleration comparisons between own and gait‐matched shoes were statistically significant (*p* = 0.011) and between own and basic shoes were approaching significance (*p* = 0.052), these differences may have been because of changes (albeit only 2.1° and 1.4° on average) in foot strike angles between shoes, indicative of larger rearfoot angles in the experimental shoes. Furthermore, the 1 g difference between shoes is equal to the 1 g SEM derived from our reliability analysis; hence, it may not be clinically meaningful. The lack of meaningful spatiotemporal or kinematic differences between gait‐matched and basic shoes indicates that shoe preferences can be influenced without large measurable biomechanical changes, in agreement with previous research [15]. This conclusion is tempered by the absence of kinetic data (e.g. ground reaction forces, joint moments).

Nearly a quarter of participants overall preferred the gait‐matched shoes above their own, indicating that they were effectively persuaded by the recommendation derived from gait analysis and favourable product description. A novelty effect of the experimental shoes may have also been present, with the cushioning of runners’ OS potentially degraded because of previous wear [44]. While diminished shoe cushioning can be difficult to detect longitudinally [45], novel shoes with fresh cushioning may be perceived as more comfortable when directly comparing between shoes [46]. The gait‐matched shoes were almost universally preferred to the basic shoes during the final ranking of shoes, supporting the VAS rating results, but both were ranked behind runners’ OS. Runners sometimes demonstrate brand affinity and prefer to use familiar footwear [11], which may explain runners’ preference for their OS. Previous research found moderate agreement between VAS ratings and a head‐to‐head preference simultaneously wearing different shoes on each foot [47]. In our study, runners compared shoes at the end of testing, which may better simulate shoe selection in running stores [12].

Runners in the present study identified that running shoe stores (in‐person), running clubs or groups and friends were the most common sources of advice that informed their running shoe selection. These sources of advice agree with previous research [6, 7, 11, 48], with a preference for interpersonal interaction. Comfort was key to running shoe selection, consistent with past online surveys [6], in‐store surveys [7] and current expert recommendations [9], in spite of being manipulable based on shoe description and deceptive recommendations.

While comfort was the most influential factor for our runners when choosing shoes (Figure 2(a)), runners ranked injury reduction as more important to footwear design (Figure 3). The importance of comfort and injury as priorities for runners is likely connected to the ideology of the ‘comfort filter’, in which comfortable shoes are posited to reduce injury incidence [49], in spite of little evidence supporting its effectiveness [5]. Participants’ motivation to run was primarily based on enjoyment and health, which may explain why the participating runners were predisposed to wanting comfortable and safe shoes, rather than prioritising performance. Industry and academic experts suggest that high‐calibre runners prioritise performance in footwear design, whereas novice and recreational runners prioritise comfort [9]. Furthermore, prior research findings indicate that runners with lower weekly mileage perceive cushioned shoes as more comfortable than minimalist ones, compared with runners who run greater weekly mileage [50]. Variations in running history and training experience may therefore influence runners’ responses to advice from a salesperson or footwear recommendation, and their overall subjective perceptions of footwear. These confounding factors were not considered in the present study. Future studies could explore how running experience, footwear habits and training background interact with footwear perceptions and selection processes.

### 4.1. Limitations

Some participants may have suspected that both shoes were the same without stating their honest thoughts in the surveys. However, this disclosing omission may reflect real‐world practices in which runners may not want to disagree with salespeople. Furthermore, runners were not blinded to the branding of the experimental shoe conditions, which also may have influenced their perceptions based on brand image. Because of resource constraints, only three sizes of experimental shoes were available. Participants falling between shoe sizes used the closest available size, which may have affected perceptual measures. Furthermore, it is possible that the experimental condition occurring between the OS trials influenced the reliability of perceptual data. Using VAS to assess perceived injury risk reduction has inherent face validity given that perceptions are subjective in nature; however, we acknowledge that perceptions of injury risk reduction may not accurately reflect real injury risk.

## 5. Conclusion

Deceptive footwear descriptions and recommendations based on a sham ‘clinical gait analysis’ affected perceptions of comfort, performance and injury risk. Compared with shoes described as ‘basic’, runners rated the same shoe described as ‘matched by running style’ as more comfortable, higher performing and less likely to cause injury. In many cases, runners rated the gait‐matched shoe similar to their OS. Since it is apparent that subjective perceptions can be manipulated based on product description, we caution runners to consider the value of choosing shoes solely based on comfort or recommendations from salespeople. However, there is no clear indication that choosing shoes based on subjective perceptions is harmful, and it may even create a positive running experience in the short term.

## Ethics Statement

The HECS Human Research Ethics Committee of the University of Waikato granted ethical approved to conduct this randomised crossover trial with repeated measures (HREC(HECS)2023#11), which followed the Declaration of Helsinki.

## Disclosure

The Running Clinic™ organisation itself was not involved in study design, data collection and analysis, decision to publish or preparation of the manuscript. This study was part of a larger doctoral thesis project examining factors that influence running footwear selection and subjective perceptions [16].

## Conflicts of Interest

Jean‐Francois Esculier is employed by The Running Clinic™, a continuing education organisation which translates scientific evidence to healthcare professionals and the public. Kim Hebert‐Losier is a speaker for The Running Clinic™.

## Author Contributions

A.F.: conceptualisation, methodology, formal analysis, investigation, data curation, writing—original draft, writing—review & editing, visualisation and project administration. K.H‐L.: conceptualisation, methodology, formal analysis, investigation, resources, data curation, writing–original draft, writing–review & editing, visualisation, supervision and project administration. J‐F.E.: conceptualisation, methodology, writing–original draft, writing–review & editing, visualisation and supervision. C.R.: conceptualization, methodology, writing–review & editing and supervision.

## Funding

The authors received no specific funding for this work.

## Data Availability

The data that support the findings of this study are available from the corresponding author upon reasonable request.
